# Tumor Escape and Progression under Immune Pressure

**DOI:** 10.1155/2012/641079

**Published:** 2012-12-19

**Authors:** Masoud H. Manjili, Nejat Egilmez, Keith L. Knutson, Senthamil R. Selvan, Julie R. Ostberg

**Affiliations:** ^1^Department of Microbiology and Immunology, Massey Cancer Center, Virginia Commonwealth University, Richmond, VA 23298, USA; ^2^Department of Microbiology and Immunology, State University of New York at Buffalo, Buffalo, NY 1421, USA; ^3^Department of Immunology, Mayo Clinic, Rochester, MN 55905, USA; ^4^Department of Medical Oncology, Thomas Jefferson University, Philadelphia, PA 19107, USA; ^5^Department of Cancer Immunotherapeutics and Tumor Immunology, Beckman Research Institute, Duarte, CA 91010, USA; ^6^Department of Hematology and Hematopoietic Cell Transplantation, Beckman Research Institute, Duarte, CA 91010, USA

Although cancers develop and progress in immunocompetent hosts, immunological therapies for cancer have been proposed as alternative or complementary approaches to more standard therapy. It was initially thought that tumors were silent to the immune system, and that breaking immunological tolerance could result in immune-mediated tumor rejection. However, we have learned that cancer patients have preexisting immune responses against their tumor antigens which, nevertheless, fail to protect them, in part because of increased activity of the immune suppressor cells such as myeloid-derived suppressor cells (MDSC). Attempts to develop combinatorial therapies by depleting suppressor cells or blocking suppressor pathways and at the same time actively inducing immune responses *in vivo* or adoptively transferring tumor-specific T cells have largely failed. Very limited success has been achieved only against melanoma, using adoptive T-cell therapy, or prostate cancer, using a vaccine which improves patient survival but has no apparent inhibitory effect on disease progression. Further progress in the immunotherapy of cancer has been halted because of a poor understanding of the cellular components of the immune responses working together in favor of or against the tumors, as well as our inability to reliably reprogram immune responses towards the most effective phenotypes against cancer. This special issue is focused on understanding the escape mechanisms that malignant cells develop to hijack antitumor immune responses as well as strategies to overcome tumor escape. Four main areas that are covered in this issue include the following.

**Opposing Functions of the Immune System in Tumor Inhibition and Tumor Progression**

Robert Schreiber proposed the term “cancer immunoediting” in order to broadly describe the dual host-protecting and tumor-sculpting actions of the immune system that not only survey for, and eliminate, nascent malignant cells but also shape neoplastic disease through equilibrium and escape mechanisms. In this issue, M. Aris et al. discuss the dual function of the immune system in controlling and promoting tumor progression in cutaneous melanoma. They propose that tumor evolution is because of a continuous feedback between tumor cells and their environment, and thus different combinatorial therapeutic approaches can be implemented according to the tumor stage. A. Amedei et al. discuss recent knowledge on the contribution of T cells in oncogenesis. They review the different types, “friend or foe,” of T-cell response in gastric cancer.

**Tumor-Associated Modulation of Immune Checkpoint Molecules**

Upon activation, T cells develop negative feedback regulatory mechanisms in order to avoid overstimulation. These include the expression of checkpoint molecules such as PD-1 and CTLA-4. T cells that recognize and respond to tumor antigens produce IFN-*γ*. A dual function of IFN-*γ* is the induction of apoptosis in target cells and upregulation of PD-L1 that interacts with PD-1 positive T cells, thereby resulting in the exhaustion of tumor-reactive T cells. Expression of CTLA-4 on activated T cells also results in T-cell anergy upon interaction with costimulatory molecules on DCs. S. Sapozink et al. describe new immunomodulatory approaches currently in the development pipeline, with focus on the novel CEACAM1 immune checkpoint, and compare its potential to the extensively described lymphocyte inhibitory targets, CTLA4 and PD-1. E. Rozali et al. provide an extensive review of the literature on the immunoregulatory role of PD-L2 in cancer-induced immune suppression and discuss the results of recent studies targeting PD-L2 in cancer. L. Cruz-Merino et al. discuss immune escape mechanisms in Hodgkin's lymphoma (HL) and summarize the clinical, histological, pathological, and biological factors in HL, with special emphasis on the improvement of prognosis and their impact on treatment strategies. L. Farnault et al. introduce various mechanisms involved in the escape of hematological malignancies from NK-cell surveillance. These include NK-cell qualitative and qualitative deficiencies that occur through modulating the inhibitory and activating stimuli.

**Tumor-Induced Immune Suppression**

Malignant cells produce cytokines and chemokines that facilitate the expansion or differentiation of immune suppressor cells such as Tregs, MDSC, and M2 macrophages. G. Zhou and H. Levitsky summarize the findings from some recent preclinical and clinical studies, focusing on how tumor cells advance their survival and expansion by hijacking therapy-induced immune effector mechanisms that would otherwise mediate their destruction. A particularly interesting notion that is touched upon involves tumor-independent treatment-induced homeostatic counter-regulation. M. Jadus et al. cover the escape mechanisms of bronchogenic lung cancer that must be overcome before they can be successfully treated. They also review the history of immunotherapy directed towards lung cancers. N. Hao et al. discuss the role of tumor-associated macrophages including M1 and M2 subsets during tumour progression and metastasis, highlighting the immunosuppressive role of M2 macrophages. V. Levina et al. investigate the role of indoleamine 2,3-dioxygenase (IDO1) in tumor escape and metastasis using 4T1 mammary carcinoma model. They show that IDO1 can not only suppress antitumour immune responses but also promote tumour cell proliferation.

**Improved Immunotherapeutic Strategies to Overcome Tumor Escape**

Immunotherapy combined with blockade of immune suppressor pathways has been developed to overcome tumor-induced immune suppression. Cornelissen et al. discuss the interplay between a dual function of the immune responses against mesothelioma which can either inhibit or stimulate tumor growth and review the challenges associated with immunotherapy. They also discuss possible strategies and opportunities to overcome tumor escape. R. Casalegno-Garduño et al. analyze the expression of the leukemia-associated antigen receptor for hyaluronan acid-mediated motility (RHAMM) in patients suffering from acute myeloid leukemia (AML) and myelodysplastic syndrome (MDS). Their results suggest that immunotherapies like peptide vaccination or adoptive transfer of RHAMM-specific T cells might improve the immune response and the clinical outcome in AML/MDS patients. S.Wallner et al. summarize the current knowledge about the negative regulatory role of Cbl-b in T-cell activation and its potential therapeutic implications for cancer immunotherapy. H. Nagai et al. demonstrate that sorafenib-induced Th1 dominance can prevent the escape of tumor cells from the host immune system in liver cirrhosis (LC) patients with advanced hepatocellular carcinoma (aHCC).

Overall, this special issue provides a well-rounded synopsis of representative research efforts addressing the issues related to “tumor escape and progression under immune pressure.”

*Masoud H. Manjili* *Nejat Egilmez* *Keith L. Knutson* *Selvan R. Senthamil* *Julie R. Ostberg*

